# High-resolution melting analysis coupled with next-generation sequencing as a simple tool for the identification of a novel somatic BRCA2 variant: a case report

**DOI:** 10.1038/s41439-018-0006-x

**Published:** 2018-06-08

**Authors:** Alessandra Costella, Rossella De Leo, Donatella Guarino, Marco D’Indinosante, Paola Concolino, Giorgia Mazzuccato, Andrea Urbani, Giovanni Scambia, Ettore Capoluongo, Anna Fagotti, Angelo Minucci

**Affiliations:** 1 Area Diagnostica di Laboratorio IRCCS, Rome, Italy; 2 Department of Obstetrics and Gynecology, Division of Gynecologic Oncology, Rome, Italy; 3 Institute of Biochemistry and Clinical Biochemistry, Teaching and Research Hospital “Agostino Gemelli” Foundation, Rome, Italy; 4 Proteomics and Metabolomics Unit, IRCCS-Santa Lucia Foundation, Rome, Italy; 5 Laboratory of Clinical Pathology and Advanced Molecular Diagnostics, Istituto Dermopatico dell’Immacolata -IRCCS, Rome, Italy

**Keywords:** High-resolution melting analysis, next-generation sequencing, novel somatic *BRCA2* variant

## Abstract

In a 72-year-old woman with no associated personal or family history of breast and/or ovarian cancers, we identified a novel somatic pathogenic *BRCA2* variant (*c.18_28delAGAGAGGCCAA*, p.Lys6Asnfs*4) using next-generation sequencing (NGS). The variant allele frequency (VAF) was 16%, and Sanger sequencing was unable to identify this variant. Adopting a high-resolution melting analysis strategy coupled with NGS, we successfully highlighted the presence of the *c.18_28delAGAGAGGCCAA* allele.

Testing *BRCA1/2 (BRCA)* genes on formalin-fixed paraffin-embedded (FFPE) or fresh tissue (FT) samples permits the simultaneous assessment of both somatic and germline variants using an easily-accessible material that is routinely available in any pathology laboratory worldwide. FFPE and FT samples are histologically heterogeneous^1^, while tumor-specific DNA contains varying proportions of contaminating DNA from normal cells.

Next-generation sequencing (NGS) methods have the potential to detect variants at low admixture levels, offering a potential solution to this challenging type of analysis^2^. Because of the poor quality of extracted DNA and to a low sequencing signal, variants in DNA from FFPE and FT sources are difficult to confirm using Sanger sequencing. Furthermore, these types of sources cannot be re-analyzed by NGS because of the small amount of DNA. To avoid considering these variants to be PCR artefacts, it is highly recommended to use alternative methodologies.

In this context, we used high-resolution melting analysis (HRMA) as a simple, cost-effective, rapid and sensitive method to confirm a novel somatic *BRCA2* variant (*c.18_28delAGAGAGGCCAA*, p.Lys6Asnfs*4) that was previously identified by NGS in a patient with high-grade serous ovarian cancer (HGSOC).

The present study involved a 72-year-old woman who presented to an oncologist with a complex right ovarian mass and elevated CA-125 level. Her gynecological history was negative. A transvaginal and transabdominal ultrasound examination revealed a multilocular solid cyst with >10 locules, papillary projections, and irregular surface with a Color Score of 4. Computed tomography of her abdomen and pelvis showed a 10 × 5 cm right ovarian mass and diffuse peritoneal enhancement, consistent with peritoneal carcinomatosis. Ovarian cancer was suspected, and the patient consented to complete surgical staging. She underwent a total abdominal hysterectomy, bilateral salpingo-oophorectomy, partial pelvic peritonectomy, and radical omentectomy 2 months after her initial presentation. The surgery was largely uncomplicated, with no significant hemostasis or coagulation issues, and optimal cytoreduction was achieved. Surgery was followed by six cycles of chemotherapy with paclitaxel and carboplatin. Written informed consent was obtained to allow *BRCA* testing to be performed after the pathological diagnosis of HGSOC was made.

DNA was extracted from FT HGSOC sections from areas with a minimum neoplastic cellularity of 70% using the MagCore Genomic DNATissue Kit by MagCore HF16 Plus (Diatech Lab Line, Jesi, Italy). The DNA concentration and quality were determined using a Qubit dsDNA HS assay (Thermo Fisher Scientific, Waltham, MA, USA).

*BRCA* analysis was performed using the Devyser BRCA kit (Devyser, Hägersten, Sweden). Sequencing reactions were carried out on the MiSeq instrument (Illumina, CA, USA). NGS data were processed using the Amplicon Suite software (SmartSeq s.r.l., Novara, Italy) with the parameters of aligning reads to the HG19 reference genome and to generate run metrics, including the depth of sequencing, total read count, and quality. In addition, *BRCA* large genomic rearrangements were also investigated as previously reported^3,4^.

Sanger sequencing and PCR-HRMA were performed on an ABI 3500 Genetic Analyzer (Applied Biosystems, Thermo Fisher Scientific) and the LightCycler® 480 Real-Time PCR System (Roche Diagnostics, Basel, Switzerland), respectively.

The WT allele is longer by 11 nucleotides compared to *c.18_28delAGAGAGGCCAA* allele; this DNA size difference allows the allele separation by capillary electrophoresis^5^. For this reason, we analyzed the PCR products on an Experion™ Automated Electrophoresis System (BioRad, Hercules, CA, USA) following the manufacturer’s instructions^5^

*BRCA* testing obtained by NGS and multiplex ligation-dependent probe amplification did not reveal any known pathogenic variants (PVs). However, the patient carried a *c.18_28delAGAGAGGCCAA* variant in exon 2 of the *BRCA2* gene. The nomenclature of the variant is based on the *BRCA2* cDNA sequence (NCBI Reference Sequence: NM_000059.3; *GRCh37*) according to the recommendations of the Human Genome Variation Society (HGVS, http://www.hgvs.org/). The average NGS read depth for the sample was ~10,000×, with a minimum and maximum depth of 2600× and 32,400×, respectively. The *c.18_28delAGAGAGGCCAA* allele showed a read depth of ~1400× on a total read count of 8700, resulting in a VAF of 16%.

Sanger sequencing, which was used to confirm the presence of the *c.18_28delAGAGAGGCCAA* allele, did not reveal this allele (Fig. 1). By contrast, high-resolution melting profiles for the patient showed a specific melting behavior compared to the FT samples (*n* = 10) that did not carry the c.*18_28delAGAGAGGCCAA* allele.

**Fig. 1:**
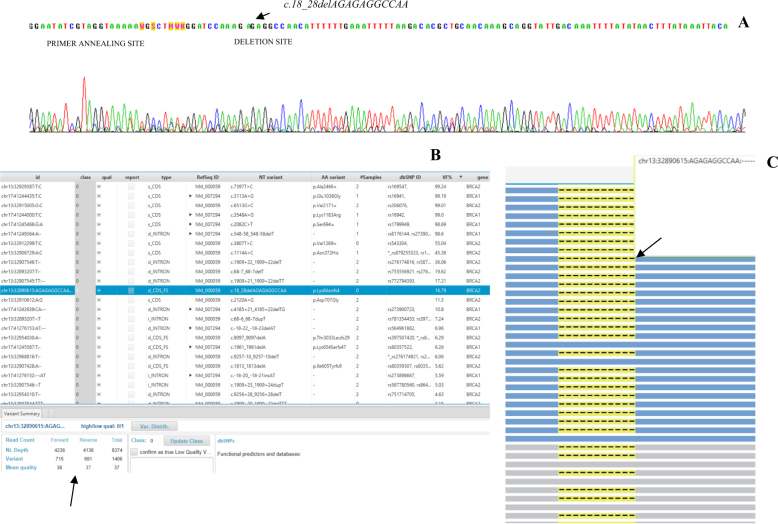
**a** Sanger sequencing was performed using the BigDye Terminator v3.1 Cycle Sequencing kit (Applied Biosystems, Thermo Fisher Scientific Inc.), and the sequencing results were analyzed using SeqScape software version 2.5 (Applied Biosystems, Thermo Fisher Scientific Inc.). The primers used for *BRCA2* exon 2 amplification and sequencing were as follows: forward (F) 5′-AGGAATATCGTAGGTAAAAATG-3′ and reverse (R) 5′-CTGGATTTATACACACATAAGG-3′. Because of the low allele frequency (16%), the presence of the *c.18_28delAGAGAGGCCAA* variant gives a sequencing signal that is indistinguishable from background noise. **b** The results of the patient’s genome obtained by NGS of the *BRCA* genes are reported; the *c.18_28delAGAGAGGCCAA* variant is highlighted (blue). Variant calling and the sequencing depth and quality were obtained using Amplicon Suite software (SmartSeq s.r.l., Novara, Italy), as highlighted by the arrow. **c** An extract of the total sequences carrying the *c.18_28delAGAGAGGCCAA* allele is shown (the deletion is indicated by the dotted line). The different colors (blue and gray) show the direction of sequencing (paired-end sequencing)

Finally, capillary electrophoresis also confirmed the presence of the *c.18_28delAGAGAGGCCAA* allele, although it was difficult to discriminate between the two alleles (Fig. 2).

**Fig. 2:**
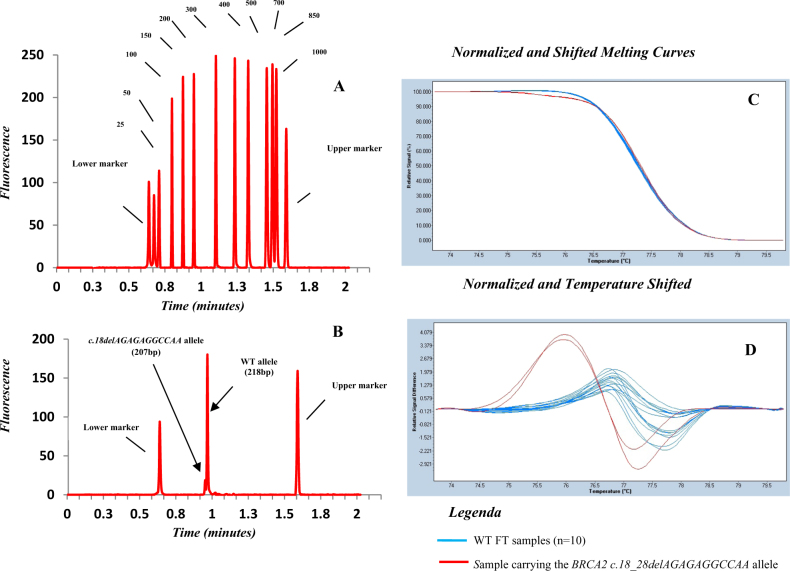
We used capillary electrophoresis to verify the presence of the c.*18_28delAGAGAGGCCAA* allele. Although it was difficult to distinguish the results because of this method’s lower resolution, amplification of the somatic mutated DNA gives two PCR products (**b**): the 218-bp peak of the WT allele and 207-bp peak corresponding to the *BRCA2 c.18_28delAGAGAGGCCAA* allele compared to the size marker (**a**). Normalized and shifted melting curves (**c**) and normalized and temperature-shifted difference plots (**d**) of the *c.18_28delAGAGAGGCCAA* allele are shown. Melting profile evaluation of the patient shows a specific melting behavior, as observed in both the normalized and the temperature-shifted and difference plots compared to the FT samples (*n* = 10) that do not carry this variant. Each experiment is reported in duplicate. The same forward primer used for sequencing was used for PCR-HRMA, while the PCR-HRMA-reverse primer was 5′-TCATTAGGGAGATACATATGGA-3′. The PCR-HRMA primers were designed using Primer3 software (http://bioinfo.ut.ee/primer3) and certified as high molecular-quality products via HPLC purification (Eurofin MWG Operon, Ebersberg, Germany)

The *c.18_28delAGAGAGGCCAA* variant was considered to be a novel variant because it was absent from 300 FT samples as well as two main variant databases: Clin Var (https://www.ncbi.nlm.nih.gov/clinvar/) and Cosmic (http://cancer.sanger.ac.uk/cosmic). This allele was also considered to be pathogenic because of its deleterious impact on the BRCA2 protein sequence.

With the implementation of treatment-focused *BRCA* testing on patients with somatic *BRCA* PVs^6^, there is an increasing clinical need for routine *BRCA* screening on DNA from FFPE and FT tumor samples. In this context, it is recommended to perform cost-effective, complete, and accurate *BRCA* gene sequencing with a sensitivity, throughput, and sample input that cannot be achieved by Sanger sequencing. In fact, Sanger approaches are not fit for detecting low VAFs, leading researchers to confuse these variants with PCR artefacts, which are indistinguishable from the background sequencing noise, and thus consider them false.

For these reasons, many diagnostic laboratories have adopted NGS technology, which offers the potential for fast, cost-efficient, and comprehensive sequencing-based testing of tumor tissue, enabling the identification of somatic *BRCA* variants.

HRMA represents a high-throughput, rapid, and inexpensive screening test for germline variants^7,8^, and because of its high sensitivity, it has also proven to be effective at identifying somatic variants^9^.

The aim of this study was to report a combined approach with NGS and HRMA to identify and confirm a novel somatic *BRCA2* variant. NGS data demonstrated a VAF of 16%, and the depth and quality of the sequencing led us to suspect that this variant was true. Using HRMA as a confirmatory test allowed us to draw up the final clinical report for this patient.

We suggest that this integrated approach can be used in diagnostic settings to improve the molecular assessment of the somatic *BRCA* status. In fact, our study provides details regarding a rapid and reliable confirmatory assay for low allele-frequency somatic *BRCA* variants that would otherwise be difficult to confirm with other molecular methods.

Best-practice guidelines for the analysis of FFPE and FT samples^10^ recommend that significant NGS findings must be replicated to ensure the reliability of the results before adopting therapeutic decisions in a clinical context.

This study demonstrates that a combined approach with NGS and HRMA allows for the reliable detection of somatic alterations affecting *BRCA* genes in FT samples, improving clinical decision-making for the treatment of HGSOC patients.

Finally, epithelial ovarian cancer treatment, which has historically been based on surgery and platinum doublet chemotherapy, is associated with a high risk of relapse and a poor prognosis for recurrent disease. In this landscape, molecular diagnosis of this somatic pathogenic *BRCA2* variant made our patient eligible for therapeutic treatments based on poly ADP ribose polymerase, which is a valuable option with promising activity in recurrent ovarian cancer patients and at the different stages of this disease.

## HGV Database

The relevant data from this Data Report are hosted at the Human Genome Variation Database at 10.6084/m9.figshare.hgv.1946
